# Menopausal hormone therapy after breast cancer: a meta-analysis and critical appraisal of the evidence

**DOI:** 10.1186/bcr1035

**Published:** 2005-05-19

**Authors:** Nananda F Col, Jung A Kim, Rowan T Chlebowski

**Affiliations:** 1 Brown Medical School and Harvard University, Providence, Rhode Island, USA; 2 Department of Nursing, Hanyang University, Seoul, Korea; 3 Harbor-UCLA Research and Education Institute, Torrance, California, USA

## Abstract

**Introduction:**

Menopausal hormone therapy (HT) is typically withheld from breast cancer survivors because of concerns about risk for recurrence. Our objectives were to estimate the effects of HT on recurrence in breast cancer survivors and to examine the reliability of these estimates.

**Methods:**

In a systematic review of the literature we identified all reports of HT use in breast cancer survivors that included comparison groups. Study design features that might affect selection of participants, detection of recurrence, and manuscript publication were assessed. The relative risks for breast cancer recurrence associated with HT were combined with random effects models.

**Results:**

Two randomized and eight observational studies included 1,316 breast cancer survivors who used HT and 2,839 nonusers. In the observational studies, HT users were younger and more commonly node negative; only two reported balanced restaging for HT and control groups. Randomized trials suggest that HT increased the risk for recurrence (relative risk 3.41, 95% confidence interval 1.59–7.33), whereas observational studies suggest that HT decreased this risk (relative risk 0.64, 95% confidence interval 0.50–0.82).

**Conclusion:**

Results from observational studies of HT conducted in breast cancer survivors are discrepant with results from randomized trials. Observational studies of HT use in breast cancer survivors have design limitations that cannot be controlled for using standard statistical methods. Therefore, the randomized clinical trial data provide the only reliable estimates of the effect of HT use on recurrence risks in breast cancer survivors.

## Introduction

Most breast cancer survivors are menopausal either at diagnosis or as a result of premature therapy-induced menopause, and they frequently experience climacteric symptoms [1]. Menopausal hormone therapy (HT), either with estrogen alone or with combined estrogen and progestin, relieves estrogen deficiency symptoms [2] but it is commonly withheld from women with diagnosed breast cancer because of concerns regarding an increased risk for recurrence [3].

The available data from observational studies indicate that use of HT is associated with increased risk for breast cancer [4]. In postmenopausal women, the randomized Women's Health Initiative HT trials found an increased risk for breast cancer with estrogen plus progestin [5] but not with unopposed estrogen [6]. An apparent reduction in risk seen during the first 2 years of combination HT was attributed to a masking of breast cancer detection, with a higher risk for more advanced breast cancers subsequently [5]. In breast cancer survivors, observational studies have consistently reported similar or lower risks for recurrence among women using HT as compared with nonusers [7], albeit with methodological weaknesses [8]; this has been interpreted as evidence of the safety or perhaps benefit of HT in women with breast cancer. However, the first large randomized trial in this population reported that HT significantly increased the risk for recurrence [9].

The objectives of this meta-analysis were to estimate the impact HT has on recurrence risk among observational and randomized studies, and to examine the reliability of these estimates.

## Materials and methods

A previous Medline search from 1966 to 1999 [7] was updated to February 2004 using the medical subject headings 'breast neoplasm', 'neoplasm recurrence', 'estrogens', 'estrogen replacement therapy', 'hormone replacement therapy', and 'estradiol', and reference lists of abstracted manuscript and protocols were reviewed. Only studies that included women with invasive breast cancer who received oral HT, that had an explicitly defined comparison group, and that reported breast cancer recurrences were included. Studies that reported overlapping or redundant data were excluded [10-16], as were those that did not adequately describe the selection or composition of control groups [17,18] or that included only topical hormones [19].

Two of the authors (NFC and JAK) independently abstracted data on the following variables: sample size, age at diagnosis and at trial induction, tumor stage, nodal status, estrogen and progesterone receptor status, disease-free interval (DFI) between initial breast cancer diagnosis and initiation of HT, type and duration of HT used, follow up after initiation of HT, and number and timing of breast cancer recurrences.

Each study was systematically reviewed for features that could introduce bias, including procedures for identifying participants, whether institutional review board approval and/or informed consent was obtained, whether risk factors for recurrence were similar at diagnosis, and whether restaging before entry (to exclude metastatic disease) and duration of follow up were similar for HT users and nonusers. Observational studies were classified as 'clinical experiences' if one or more study authors provided health care to the cohort with potential participation in the decision to use HT.

When not reported, the follow up after HT initiation was assumed to equal the duration of HT use. Any second breast cancer event (local, regional, or distant recurrence or invasive cancer in either breast) was treated as a recurrence because studies did not consistently make these distinctions.

Relative risk (RR) and 95% confidence interval (CI) were calculated for each study for the recurrence rate and mortality rate among HT users and nonusers. A random effects model was used to estimate the combined RR for randomized and observational studies using Meta-Analyst [13].

## Results

Ten studies were identified, including a total of 1,316 breast cancer survivors who used HT and 2,839 who did not. Of these 10 studies, two were unblinded randomized controlled trials without placebo arms [9,20], one began as a randomized trial but was reported as an observational study and is considered as such here, and seven were observational studies.

### Summary of randomized trials

Both randomized trials were conducted in Europe (one in England and one in Sweden). They involved a total of 445 patients with a mean age of 55.5 years, a mean DFI of 33.2 months, a duration of HT use of 19.9 months, and a mean follow-up period after HT initiation of 25.2 months (Table 1). A total of 36 recurrences and nine deaths occurred during this time in these trials; the pooled RR for the two randomized trials was 3.41 (95% CI 1.59–7.33).

### Summary of observational studies

Of the eight observational studies, six were clinical experiences [22-27]. The eight studies involved a total of 3710 patients with a mean age of 59.7 years, a mean DFI of 49.2 months, a duration of HT use of 28 months, and a mean follow-up period after HT initiation of 57.1 months (Table 1). A combined total of 552 recurrences (109 among HT users) and 460 deaths (51 among HT users) occurred in these trials. The pooled RR for the observational studies was 0.64 (95% CI 0.50–0.82).

### All studies

Most studies included both combination HT and unopposed estrogens without stratifying risk estimates according to preparation. Three of the observational studies [22,24,25] reported obtaining informed consent but only from women who used HT. Three studies [20,24,26] reported similar restaging for treatment and control groups at the beginning of the observation period, although one of these [26] did not report whether those found to have occult metastasis were excluded. Not all studies reported the DFI for the control groups, but several reported matching control individuals according to DFI [22,27]. Prognostic factors for HT users and nonusers differed in most studies (Table 1). On average, HT users were more than 3 years younger than nonusers and were more likely to be node negative. The average duration of HT use was 26.6 months, with an average duration of follow up after initiation of HT of 53 months. The mean DFI was 36.9 months for HT users and 55.6 for nonusers.

**Figure 1:**
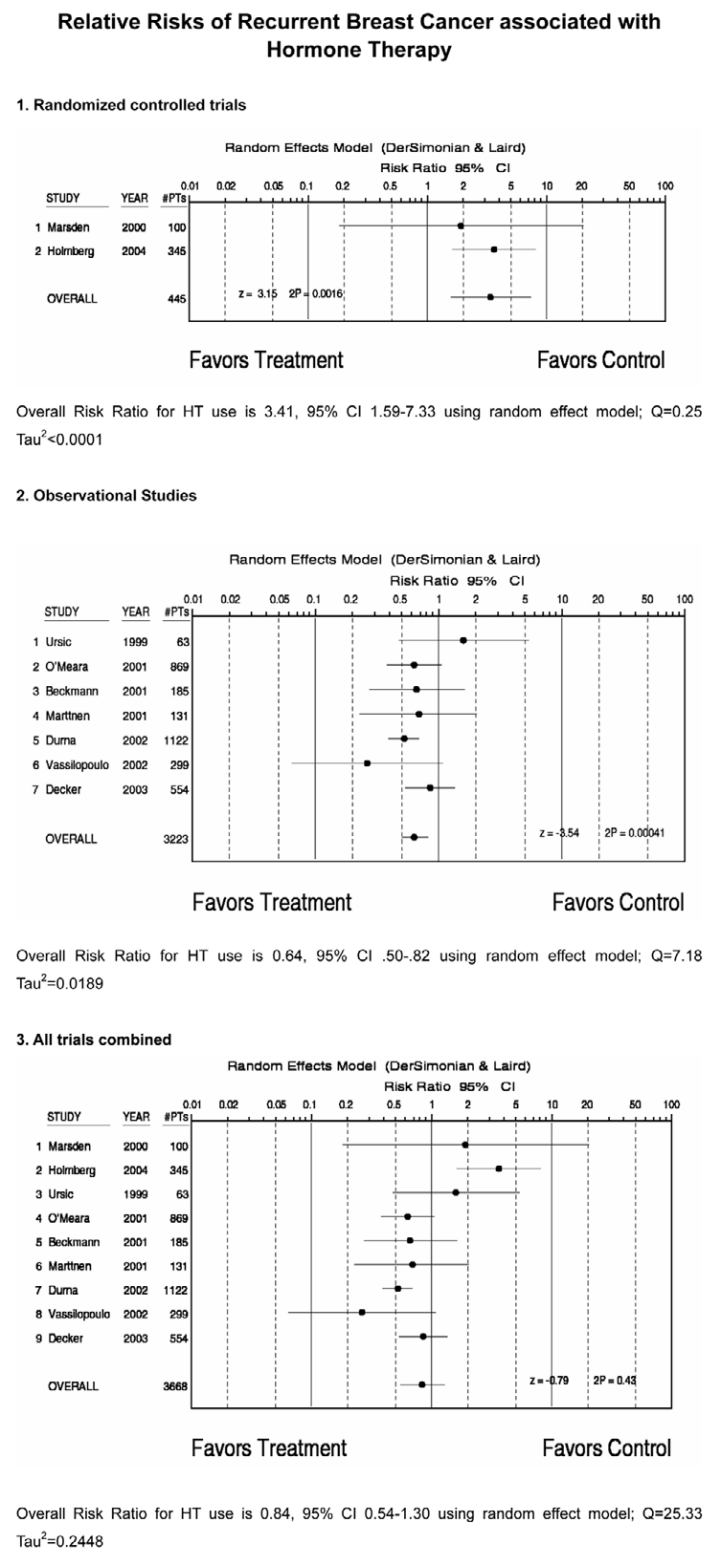
Relative risks for recurrent breast cancer associated with hormone therapy (HT). Each black circle indicates the relative risk for recurrent breast cancer; the horizontal lines indicate the 95% confidence interval (CI). The top portion of the figure describes randomized controlled trials, the middle portion describes observational studies, and the bottom portion describes all trials combined.

Among the 1,191 HT users in nine studies reporting recurrences, 137 (11.7%) experienced a recurrence of their breast cancer during follow up. Among the 2,477 nonusers in these studies, 451 (18.2%) had a recurrence. The average annual recurrence rate was 3.3% (range 0.6–7.1%), with substantially higher rates in the randomized trials. Combining all studies yielded a RR for recurrence of 0.84 (95% CI 0.54–1.3; Fig. 1), with statistically significant heterogeneity (Q = 25.3).

## Discussion

Estimates from observational studies of HT among breast cancer survivors suggest that HT prevents breast cancer recurrence, whereas estimates from randomized trials suggest the opposite. Because of statistically significant heterogeneity, these estimates should not be combined. Although all of the trials included in our analyses contained methodological weaknesses, the nonrandomized studies had design features that could introduce selection, reporting, and/or publication biases. The selection of healthier women to begin HT, the benefit of restaging before initiation of HT, the short duration of HT exposure and follow up, the potential effects of HT on mammograms that could obscure the diagnosis of recurrent or new breast cancers, and publication bias favoring publication and/or completion of studies reporting a protective effect of HT could explain the apparent protective effect of short-term HT on recurrence among breast cancer survivors in these studies.

Systematic serial restaging with blood tests and imaging during follow up is no longer generally recommended. However, their use detects breast cancer recurrence earlier. Balanced restaging was defined in only two out of seven observational studies. If breast cancer survivors contemplating HT use were more likely to have restaging, then the imbalance could account for the apparent protective effect of HT in observational studies. Although the description of prognostic factors was rarely complete, HT users in observational studies were younger and had more favorable prognostic profiles than did control individuals. This process also selected women with severe vasomotor symptoms, who have lower estradiol and testosterone levels; higher levels of these hormones have been associated with increased breast cancer risk. As a result, it is possible that women who were more likely to be offered HT [20] had lower recurrence risks. It is important to note that the majority of observational studies included in these analyses were not designed as observational studies from the start but rather as clinical experiences. Had these observational studies been more rigorously designed, using modern epidemiological techniques, many of these biases could have been minimized.

The adverse effect of combined HT on mammographic breast cancer detection [5] might have affected recurrence detection. Both recurrent and new breast cancers, which account for 10–20% of cancer events in women with prior lumpectomy, could have falsely appeared lower in HT users because of HT-related interference with mammographic diagnosis. However, this factor is probably not large, given the sharp increase in risk observed even after short-term HT use in randomized trials [36] and that the increase in risk pertained to distant as well as local recurrences.

The randomized trial reported by Holmberg and colleagues [9] overcomes many of the shortcomings of observational studies and provides the best available data on the impact of HT in breast cancer survivors. Although their unblinded design and lack of a placebo group could result in selective attrition, follow-up rates were comparable among HT users and nonusers. These investigators also reported summary interim analyses of a similar randomized trial, the Stockholm trial, with a relative hazard ratio of 0.82 (95% CI 0.35–1.9). This trial was not included in this analysis because its findings have not yet been reported in full; the reasons for its discrepant findings are unclear at this time.

## Conclusion

Observational studies of HT use in breast cancer survivors have design limitations that cannot be controlled for using standard statistical methods and hence should be considered essentially uninformative with respect to the safety of HT use in breast cancer survivors. Only randomized clinical trials are likely to provide reliable estimates of the effect of HT use in this setting.

## Abbreviations

CI = confidence interval; DFI = disease-free interval; HT = hormone therapy; RR = relative risk.

## Competing interests

The author(s) declare that they have no competing interests.

## Authors' contributions

NC conceived the study (with RC), designed the study, reviewed the source studies, abstracted data, drafted the paper, and supervised the statistical analyses. JK participated in the design of the study and reviewed the source studies, abstracted data, carried out the meta-analysis, and helped to draft the manuscript. RC conceived of the study (with NC), designed the analysis, participated in its coordination, and helped to draft the manuscript. All authors read and approved the final manuscript.

## Figures and Tables

**Table 1:** Characteristics of 1316 users and 2839 nonusers of hormone therapy

Study	Treatment	*n*	Mean age (years)	Stage	Nodal status	ER status	PgR status	Mean DFI before HT (months)	Estrogen alone (%)	Mean duration of HT (months)	Mean follow-up after HT (months)	Recurrences (*n*)	Deaths, all cause (*n*)	Deaths, primary tumor (*n*)
Randomized trials														
Marsden *et al*. (2000; *n *= 100) [20]	HT	51^a^	58^b^	NR	NR	NR	NR	40^b^	NR	6	NR	2	NR	NR
	No HT	49^a^	55^b^	NR	NR	NR	NR	36^b^			NR	1	NR	NR
Holmberg *et al*. (2004; *n *= 345) [9]	HT	174	55.5	NR	25.9% (38) positive	86 positive^c^	NR	31.2^b^	NR	24	25.2^b^	26	5	3
	No HT	171	55.0	NR	21.4% (31) positive	73 positive^c^	NR	32.4^b^			25.2^b^	7	4	4
Observational studies														
Ursic-Vrscaj and Bebar (1999; *n *= 63) [27]	HT	21^d^	47^b^	1 G1 10 G2 7 G3	14 negative, 7 positive	5 positive, 16 negative	8 positive, 13 negative	62	4.8	28	38^g^	4	0^g^	0
	No HT	42^d^	48.2	7 G1 17 G2 11 G3	28 negative 14 positive	18 positive, 22 negative	22 positive, 18 negative	NR			38^g^	5	1^g^	1
DiSaia *et al*. (2000; *n *= 487) [22]	HT	125	55.7	17 DCIS 52 stage I 27 stage II 10 stage III 1 stage IV	NR	NR	NR	46^b^	28	22^b^	92.1^g^	NR	4^g^	NR
	No HT	362	55.9	NR	NR	NR	NR	NR			90.6^g^	NR	57^g^	NR
O'Meara *et al*. (2001; *n *= 869) [36]	HT	174^d^	63.6^e^	91 stage I 51 stage II 20 stage I/II 10 stage III 2 stage II/III	128 negative, 31 positive	84 positive, 39 negative	71 positive, 45 negative	47.7^e^	79	15^b^	44.4^b,f^	16	17	5
	No HT	695^d^	63.6^e^	403 stage I 246 stage II 3 stage I/II 42 stage III 1 stage II/III	470 negative, 175 positive	409 positive, 137 negative	311 positive, 206 negative	47.7^e^			44.4^b,f^	101	115	59
Beckmann *et al*. (2001; *n *= 185) [24]	HT	64	NA	37 T1 19 T2 8 T3/4	44 negative, 20 positive	31 positive, 33 negative	34 positive, 30 negative	0	NA	33^b^	37^b^	6	4	NR
	No HT	121	NA	62 T1 42 T2 17 T3/4	76 negative, 45 positive	48 positive, 73 negative	48 positive, 73 negative	0			42^b^	17	15	NR
Marttunnen *et al*. (2001; *n *= 131) [26]	HT	88	53.4	3 DCIS 67 T1 17 T2 1 T3	72 negative, 10 positive	57 positive, 15 negative	54 positive^g^, 13 negative^g^	50.4	38.6	30	30	7	2	2
	No HT	43	52.8	1 DCIS 29 T1 11 T2 2 T3	30 negative, 13 positive	29 positive, 9 negative	30 positive^g^, 7 negative^g^	50.4			31.2	5	3	3
Durna *et al*. (2002; *n *= 1122) [23]	HT	286	56.8^b^	180 stage I 64 stage II 22 stage III/IV	NA	NR	NR	12^b^	5.9	21^b^	69.6^b^	44	16	13
	No HT	836	64.7^b^	470 stage I 191 stage II 120 stage III/IV	NA	NR	NR	NR			61.2^b^	247	199	122
Vassilopoulou-Sellin *et al*. (2002; *n *= 299) [21]	HT	56^h^	56^b^	9 <1 cm 30 1–2.5 cm 15 >2.5 cm	35 negative, 13 1–3, 6 >3	37 negative	NR	105.6	100	30 >5 years, 20 2–5 years, 6 2 years	71	2	1	0
	No HT	243^h^	53^b^	38 <1 cm 134 1–2.5 cm 67 >2.5 cm	133 negative, 70 1–3, 33 >3	164 negative	NR	99.6			NR	33	2	1
Decker *et al*. (2003; *n *= 554) [25]	HT	277	57.4^b^	84 DCIS 124 stage I 47 stage IIA 19 stage IIB 3 stage IIIA	NR	100 positive, 54 negative	63 positive, 46 negative	43.3	48.7	44.4	49.7	30	7	5
	No HT	277	59.0^b^	84 DCIS 124 stage I 47 stage IIA 19 stage IIB 3 stage IIIA	NR	121 positive, 35 negative	73 positive, 42 negative	NR			45.6	35	17	9
Summary														
Randomized trials	HT	225	56.07		38 positive	86 positive		33.19		19.92	25.20	28	5	3
	No HT	220	55.00		31 positive	73 positive		33.20			25.20	8	4	4
Observational studies	HT	1091	56.98		293 negative, 87 positive	277 positive, 194 negative	230 positive, 147 negative	37.70	40.4	28.02	57.46	109	51	25
	No HT	2619	60.87		737 negative, 350 positive	625 positive, 440 negative	484 positive, 346 negative	54.01			57.02	443	409	195
All combined	HT	1316	56.82		293 negative, 125 positive	363 positive, 194 negative	230 positive, 147 negative	36.93	40.4	26.58	53.03	137	56	28
	No HT	2839	60.39		737 negative, 381 positive	698 positive, 440 negative	484 positive, 346 negative	50.55			54.88	451	413	199

^a^Excluding stage III/IV patients. ^b^Median value. ^c^Refers to hormone receptor status; specific data concerning estrogen receptor (ER) and progesterone receptor (PgR) status were not reported. ^d^Excluding patients with ductal carcinoma *in situ *(DCIS). ^e^Weighted mean. ^f^For recurrence only; follow-up for mortality was 55.2 months. ^g^Personal communication. ^h^Excluding DCIS, stages III and IV, and ER-positive patients. DFI, disease-free interval; HT, hormone therapy; NA, not able to calculate; NR, not reported.
